# SVM-based prediction of caspase substrate cleavage sites

**DOI:** 10.1186/1471-2105-7-S5-S14

**Published:** 2006-12-18

**Authors:** Lawrence JK Wee, Tin Wee Tan, Shoba Ranganathan

**Affiliations:** 1 Department of Biochemistry, Yong Loo Lin School of Medicine, National University of Singapore, Singapore; 2 Department of Chemistry and Biomolecular Sciences & Biotechnology Research Institute, Macquarie University, Sydney, Australia

## Abstract

**Background:**

Caspases belong to a class of cysteine proteases which function as critical effectors in apoptosis and inflammation by cleaving substrates immediately after unique sites. Prediction of such cleavage sites will complement structural and functional studies on substrates cleavage as well as discovery of new substrates. Recently, different computational methods have been developed to predict the cleavage sites of caspase substrates with varying degrees of success. As the support vector machines (SVM) algorithm has been shown to be useful in several biological classification problems, we have implemented an SVM-based method to investigate its applicability to this domain.

**Results:**

A set of unique caspase substrates cleavage sites were obtained from literature and used for evaluating the SVM method. Datasets containing (i) the tetrapeptide cleavage sites, (ii) the tetrapeptide cleavage sites, augmented by two adjacent residues, P_1_' and P_2_' amino acids and (iii) the tetrapeptide cleavage sites with ten additional upstream and downstream flanking sequences (where available) were tested. The SVM method achieved an accuracy ranging from 81.25% to 97.92% on independent test sets. The SVM method successfully predicted the cleavage of a novel caspase substrate and its mutants.

**Conclusion:**

This study presents an SVM approach for predicting caspase substrate cleavage sites based on the cleavage sites and the downstream and upstream flanking sequences. The method shows an improvement over existing methods and may be useful for predicting hitherto undiscovered cleavage sites.

## Background

Caspases belong to a unique class of cysteine proteases which function as critical effectors of apoptosis, inflammation and other important cellular processes such as cell proliferation, cell differentiation, cell migration and receptor internalization [1-3]. Caspases contain a cysteine residue at the active site and cleave substrates at specific tetrapeptide sites (denoted P_4_-P_3_-P_2_-P_1_) with a highly conserved aspartate (D) at the P_1 _position [4]. To date at least 14 mammalian caspases have been discovered and they can be grouped into three classes based on their preferential tetrapeptide specificities [5]. Group I caspases (-1, -4 and -5) recognize the sequence (W/L)EHD; Group II caspases (-2, -3 and -7) prefer the sequence DEXD; while Group III caspases (-6, -8, -9 and -10) cleave proteins with the sequence (L/V)E(T/H)D.

As reviewed in Earnshaw *et al*. [6] and Fischer *et al*. [7], substrates of caspases belong to a myriad of protein classes such as structural elements of cytoplasm and nucleus, components of the DNA repair machinery, protein kinases, GTPases and viral structural proteins. Although more than 280 caspase substrates have been discovered to date, it is possible that several more remain undetected [6,7]. The identification and characterization of caspase substrates are critical for deepening our understanding of the role of these enzymes in the various cellular pathways. However, the accurate detection of caspase cleavage sites in target proteins requires complex and time consuming *in vivo *and *in vitro *experiments. Given the readily available sequence data in public databases, a useful alternative is to conduct *in silico *screening for potential cleavage sites among proteins. While the preferential cleavage specificities may be useful here, recently identified substrates have shown significant variation in their cleavage sites [7]. Therefore, the development of computational tools to accurately capture complex sequence patterns and to automate the identification of new cleavage sites would be valuable.

A number of caspase substrate cleavage prediction methods currently exist. The pioneering work began with PeptideCutter, a proteases substrates cleavage prediction server for various families of proteases. Due to the scarcity of experimental data, PeptideCutter was based only on the preferential cleavage specificities of certain caspases [8]. Lohmuller *et al*. [9] developed the peptidase substrate prediction tool (PEPS) based on position specific scoring matrices (PSSM) for cathepsin B, cathepsin L and caspase-3 substrates. While useful, the utility of these tools is limited as they were built on a small dataset of cleavage sites and the cleavage specificities are confined to certain caspases alone, rather than the entire family. In recent years, the exponential discovery and characterization of new substrates and their cleavage sites [7] enabled the development of more effective algorithmic tools. Garay-Malpartida *et al*. [10] developed the CasPredictor software which exhibited an improvement over previous methods with an accuracy of 81% on a dataset of 137 experimentally verified cleavage sites. The CasPredictor software uses an algorithm which analyzes the cleavage sites for amino acid substitution, amino acid frequency and the presence of 'PEST' sequences [11,12] in the vicinity of the cleavage site (flanking 10–15 residues). The GraBCas software by Backes *et al*. [13] advanced the previous PSSM-based methods by including an updated set of caspase cleavage specificities based on the work by Thornberry *et al*. [5], and observing conservation at P_1_' and even P_2_' positions. Yang [14] experimented with different neural networks for predicting cleavage sites such as single-layer perceptrons, multi-layer perceptrons and the Bayesian bio-basis function neural networks. They achieved an accuracy of 97% using the Bayesian bio-basis function neural network with two Gaussian distributions. In the same study, the SVM method was tested and was found to give excellent results. However, Yang used a small dataset of 13 sequences and the method is not available for testing.

In this study, we have developed a support vector machine (SVM) system to predict the caspase substrate cleavage sites. First introduced by Cortes and Vapnik [15], the SVM method is a relatively new sub-branch of the machine learning algorithms. SVM has been shown to perform well in diverse computational biology applications such as the prediction of protein secondary structure [16-18]; protein fold [19]; protein quaternary structure [20]; protein homology [21]; protein-protein interaction sites [22]; protein domains [23], HIV protease cleavage sites [24] and T-cell epitopes [25]. It is also used in the classification and validation of cancer tissue samples [26] and microarray expression data [27]. Other applications of SVMs in biology have been reviewed by Byvatov and Schneider [28], and Yang [29]. We have compiled an extensive dataset of unique (non-redundant) cleavage sites to validate the SVM method and to further the development of other computational tools. Using various statistical metrics, we have shown that the SVM method is a rigorous and effective approach for predicting cleavage sites of caspase substrates.

## Results and Discussion

The prediction of caspase substrate cleavage sites is important for our in-depth understanding of the protease-substrate interaction as well as in identifying new caspases substrates. Since the publication of the preferential tetrapeptide specificities by Thornberry *et al*. [5], many more caspase substrates have been discovered and the reported cleavage sites have been shown to vary considerably from the preferred sequences [7]. Artificial intelligence-based techniques such as SVM and the neural network are elegant approaches for the extraction of complex patterns from biological sequence data. As the SVM methodology was successfully applied in several biological problems, we investigated the utility of the SVM approach in predicting the cleavage sites of caspase substrates.

**Figure 1:**
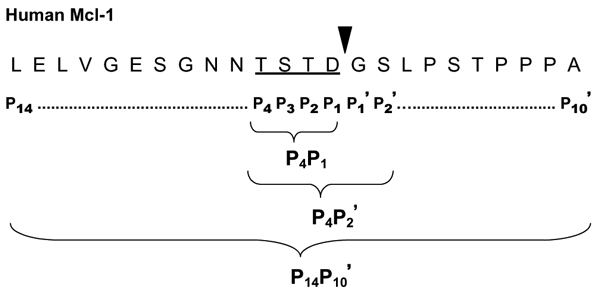
**Different subsequence segments for SVM training and testing**. For human Mcl-1 [Swiss-Prot:Q07820], a sequence window of 24 amino acids in length centred on the tetrapeptide cleavage site, TSTD (underlined) is shown. Amino acids to the left of the scissile bond (shown by the inverted triangle) are labelled from P_1 _(D) to P_14 _(L). Amino acids to the right of the scissile bond are labelled from P_1_' (G) to P_10_' (A). Curly brackets indicate the subsequence segments extracted for SVM implementation. The sequences spanning P_4 _to P_1 _(TSTD), P_4 _to P_2_' (TSTDGS) and P_14 _to P_10_' (LELVGEGSNNTSTDGSLPSTPPPA) are labelled as P_4_P_1_, P_4_P_2_' and P_14_P_10_' respectively.

Based on the work by Fischer *et al*. and through our own data mining efforts, we have compiled a database of experimentally determined caspase substrates annotated with their cleavage sites. We have obtained a set of 195 unique cleavage sites from Fischer *et al*. and 24 unique cleavage sites from recently discovered caspase substrates reported in literature but were not detailed in Fischer *et al*. The 195 sequences were used for training the SVM classifier while the 24 sequences were used for testing the effectiveness of the SVM method. As there were no experimentally reported non-cleavage sites for caspases, we extracted tetrapeptide sequences at random positions (not including the cleavage sites) on experimentally determined caspase substrates. One non-cleavage site was extracted for every cleavage site on the same substrate. The assumption that an intuitively large proportion of tetrapeptide sequences other than the cleavage site(s) on the same substrate should not be recognized and cleaved by caspases justifies the use of these sequences as non-cleavage sites. An equal number of these non-cleavage sites were extracted to match the cleavage sites. Together, a primary dataset consisting of the tetrapeptide cleavage sites (positive examples) and non-cleavage sites (negative examples) was constructed and designated as the P_4_P_1 _dataset (Figure 1).

Previously, Backes *et al*. [13] and Garay-Malpartida *et al*. [10] suggested that residues adjacent to the cleavage site may influence substrate cleavage. Backes *et al*. reported the high occurrence of specific amino acids at P_1_' for caspase-3 and P_1_' and P_2_' for granzyme B, a serine protease involved in apoptosis and in immune response [30]. Garay-Malpartida *et al*. reported that a sizeable proportion of cleavage sites are localized within 'PEST' regions, of which have been suggested to label proteins for protease degradation. PEST regions are defined as sequence segments enriched with proline (P), glutamate (E), aspartate (D), serine (S) and threonine (T) residues [11,12]. Therefore, to investigate the influence of the adjacent sequences on substrate cleavage, we further constructed a dataset containing tetrapeptide sequences with the P_1_' and P_2_' residues and a dataset containing tetrapeptide sequences flanked by ten residues on either side of the cleavage site. These datasets were designated as P_4_P_2_' and P_14_P_10_' respectively (Figure 1). The longer sequence segments would encapsulate the information contained in the critical tetrapeptide sequences as well as the P_1_' and P_2_' amino acids and other residues adjacent to the cleavage sites.

**Figure 2:**
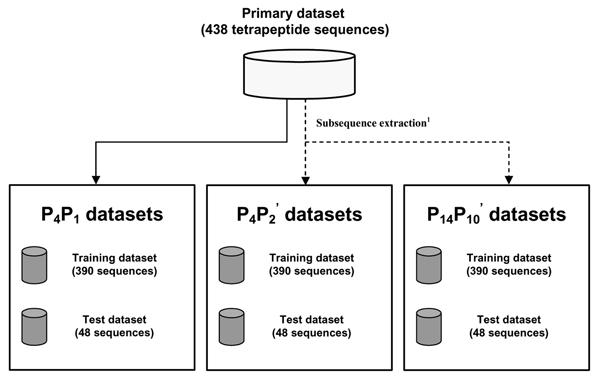
**Schematic layout of the datasets used for SVM training and testing**. The primary dataset consist of non-redundant tetrapeptide caspase substrate cleavage sites obtained from literature (see Additional File 1) and an equal number of non-cleavage sites. ^1^The P_4_P_1 _sequences consist of all the sequences in the primary tetrapeptide cleavage site dataset. P_4_P_2_' and P_14 _P_10_' datasets were derived by extracting subsequence segments from the parent protein chains in the vicinity of the tetrapeptide cleavage sites, as shown in Figure 1. All datasets contain equal number of positive and negative examples.

Next, we divided the P_4_P_1_, P_4_P_2_' and P_14_P_10_' datasets into training and test datasets (Figure 2). The training datasets were used for optimizing the SVM parameters and for training the SVM classifier, while the test datasets were used for evaluating the SVM method. We have chosen the RBF kernel which requires parameters *γ *and *C *to be optimized. Using 10-fold cross-validation, the parameters γ and *C *were optimized at 0.01 and 100 (for P_4_P_1 _training dataset) and 0.1 and 100 (for both P_4_P_2_' and P_14_P_10_' training datasets). For each of P_4_P_1_, P_4_P_2_' and P_14_P_10_' training datasets, an overall accuracy of 98.97% was obtained during the cross-validation.

While the reported accuracy on the training datasets may indicate the effectiveness of a prediction method, it may not accurately portray how the method will perform on novel, hitherto undiscovered cleavage sites. Therefore, testing the SVM methodology on independent out-of-sample datasets, not used in the cross-validation is critical. Here, we applied the SVM classifiers, trained separately using the entire training datasets from the P_4_P_1_, P_4_P_2_' and P_14_P_10_' datasets with the optimized *γ *and C parameters, on the respective test datasets and evaluated the results. As shown in Table 1, for the P_4_P_1 _test dataset, the SVM method obtained an accuracy of 95.83% using the RBF kernel with *γ *= 0.01 and C = 100. For both the P_4_P_2_' and P_14_P_10_' test datasets, the SVM method obtained an accuracy of 97.92% using the RBF kernel with *γ *= 0.1 and C = 100.

Our analysis on the training and test datasets indicated a large percentage of cleavage sites with the XXXD motif (~98%) and a very small percentage of cleavage sites with a non-canonical XXXE motif (~2%). While experimental cleavage site specificities reported in Thornberry *et al*. suggest most, if not all, sequences to conform to the XXXD motif [5], the inclusion of a large proportion of these sequences in the development of the SVM system could lead to over-training of the classifier and confound the results obtained with different sequence representations. To mitigate this possibility, we further constructed datasets identical to P_4_P_1_, P_4_P_2_' and P_14_P_10_' datasets, but with the P_1 _residue removed in all the sequences (labelled as P_4_P_1_(-D), P_4_P_2_' (-D) and P_14_P_10_' (-D) datasets respectively). These datasets were further divided into training and test sets and SVM parameters were optimized in the manner as reported for the original P_4_P_1_, P_4_P_2_' and P_14_P_10_' datasets. The trained SVM classifiers were tested on the respective test datasets. As shown in Table 1, the SVM method obtained an accuracy of 81.25% for the P_4_P_1 _(-D) test dataset. The performance of the SVM improved significantly when tested on P_4_P_2_' (-D) and P_14_P_10_' (-D) datasets as accuracy readings of 89.58% and 93.75% were obtained respectively. While the accuracy on all (-D) test datasets were lower compared to the corresponding original datasets, a larger degree of improvement was observed when the longer sequence representations were used, as evidenced by the greater spread in both the accuracy and sensitivity readings for the P_4_P_1_(-D), P_4_P_2_' (-D) and P_14_P_10_' (-D) datasets. An analysis of the misclassified sequences showed that cleavage sites such as CLLD^2193 ^from Notch1 [Swiss-Prot:P46531] and PEVD^142 ^from p23 co-chaperone [Swiss-Prot:Q15185], which differ markedly from reported tetrapeptide specificities [5], were misclassified by the P_4_P_1 _(-D)-trained SVM, but were correctly predicted when the P_4_P_2_' (-D) and P_14_P_10_' (-D) datasets were used. Also, the SVM trained with the P_4_P_1 _(-D) and P_4_P_2_' (-D) datasets failed to correctly classify the non-canonical cleavage site VQPE^205 ^from DIAP1 [Swiss-Prot:Q24306], but correctly predicted the cleavage site when trained with the P_14_P_10_' (-D) dataset. These results suggest that the SVM trained with the (-D) datasets may be useful for identifying hitherto undiscovered cleavage sites while circumventing the problem of overtraining due to the high percentage of "XXXD" cleavage sites in the training datasets. The results also provided further evidence for the suggestion that the P_1_', P_2_' and residues further upstream and downstream of the cleavage site may influence substrate cleavage, and by accounting for these flanking sequences, the SVM performance can be improved. It was also shown that the SVM method can be extended to predict cleavage sites with residues other than the canonical aspartate (D) at P_1_. While the occurrence of the non-canonical cleavage sites remains proportionately small, it does imply that the sampling space is not limited to the XXXD motif for cleavage sites. Consequently, the ability to predict these non-canonical cleavage sites will be a useful complement to existing computational methods which assumes the consensus XXXD motif as the basis for their algorithms.

As other methods were not readily accessible, we were only able to compare the GraBCas method on our datasets. Since the GraBCas method primarily focuses on the tetrapeptide motif, we have applied it to the P_4_P_1 _training dataset alone. As the GraBCas method can only be applied to potential cleavage sites with aspartate (D) at the P_1 _position, we scored the positive sequences in the P_4_P_1 _training dataset with the GraBCas matrix values for the different caspases, selected the highest score and checked for the percentage of correctly predicted cleavage sites *(or Sensitivity, SE) *against a series of cut-off scores. As shown in Table 2, the sensitivity values declined steadily from 87.43% to 19.76% as the cutoff values were progressively increased (0.1, 1, 5, 10, 20). We have also tested the GraBCas method on the positive sequences in the P_4_P_1 _test dataset. As there were no recommended cut-off scores for predicting the cleavage sites, we chose the cut-off score of 0.1, which was used for the granzyme B cleavage site prediction as reported in Backes *et al*. [13]. At the cut-off score of 0.1, GraBCas predicted only 16 out of 24 cleavage sites correctly *(SE = 66.67%)*.

Finally, to investigate how the SVM approach can complement experimental work on caspase substrate cleavage, we applied the SVM approach to predict the caspase-mediated cleavage of an anti-apoptotic protein, Livin [Swiss-Prot:Q96CA5] and its mutant sequences as reported in Yan *et al*. [31], based on the prediction of the caspase cleavage sites. As shown in Table 3, the experimental cleavage of wild type human Livin and its deletion mutants were compared to the results predicted by the SVM trained with the P_14_P_10_' (-D) dataset. With the exception of the LE Δ52–61, Δ51–53 and Δ53–61 mutants, all other sequences were correctly predicted to be cleaved or not cleaved by caspases as indicated. For the LE Δ52–61 and Δ51–53 mutants, the flanking sequences upstream and downstream of the cleavage site were likely to have influenced cleavage of the substrates, as predicted by the SVM. However, cleavage of substrates was prevented due to the absence of the Asp at P_1 _(DHVD^52^). While the SVM predicted the cleavage of Δ53–61 mutant, it was proposed by Yan *et al*. that the deleted residues might have led to the distortion of the structure of a neighboring domain or affected its signaling function, which subsequently inhibited the substrate cleavage through downstream signaling. These findings suggest that the SVM-based prediction of caspase substrate cleavage sites might be helpful in identifying potential caspase substrates.

## Conclusion

In conclusion, we have compiled an extensive dataset of caspase substrates cleavage sites as reported in the literature for the development and validation of other computational tools. We have rigorously tested the SVM approach for recognizing the cleavage sites of these substrates. Our results show that the SVM method is complementary to existing methods, if not more effective. The prediction accuracy can also be improved by accounting for sequences at the P_1_' and P_2_' positions and further upstream and downstream of the cleavage site. In addition, the SVM method may be useful for predicting the non-canonical cleavage sites lacking aspartate (D) at the P_1 _position, such as those found in DIAP1 and other proteins as reported in literature [7]. As the substrate proteins used in the present method are derived from a variety of organisms (human, mouse, rat, fruit fly, cow, chicken, frog, worm and viruses) and are cleaved by various caspases (caspase-1,-3, -6, -7, -8, -9, -12, -13 and -14), our methodology is applicable to the detection of cleavage sites in substrates from various organisms and is not caspase-specific.

Together with existing computational tools, our method will complement on-going experimental efforts in identifying new caspase substrates and further our understanding of the biochemistry of caspase substrate cleavage. This knowledge will be helpful for resolving the larger role of these proteases and their targets in critical processes like apoptosis and inflammation. As more information about caspases and their substrates becomes available, we will update and improve the performance of our methodology.

## Methods

### Datasets

Our primary dataset contains 438 unique sequences (219 cleavage sites and 219 non-cleavage sites). Of the 219 cleavage sites, 195 were obtained from Fischer *et al*. and 24 from literature search. Besides the tetrapeptide cleavage site sequences, subsequence segments of varying lengths centered on the tetrapeptide cleavage sites were extracted as shown in Figure 1. In total, three groups of sequences were obtained: tetrapeptide cleavage sequences (henceforth termed as the P_4_P_1 _sequences), tetrapeptide cleavage sequences with the next two residues, P_1_' and P_2_' residues (P_4_P_2_'sequences), and tetrapeptide sequences with upstream residues up to P_14 _and downstream residues up to P_10_' (P_14_P_10_' sequences). The cleavage sites and the corresponding subsequences were designated as positive examples for the SVM training and testing.

The 219 non-cleavage sites were obtained by extracting tetrapeptide sequences at random positions (not including the cleavage sites) on caspase substrates. One non-cleavage site was extracted for every cleavage site on the same substrate. Subsequence segments centered on these non-cleavage sites were also extracted in the manner reported earlier. The non-cleavage sites and the corresponding subsequences were designated as negative examples for SVM training and testing. Together, the positive and negative examples in the three group of sequences were designated as the P_4_P_1_, P_4_P_2_' and P_14_P_10_' datasets respectively. Each of these datasets were further divided in the following manner (Figure 2):

#### 1. Training datasets

Training datasets were used for optimizing the SVM parameters and for training the SVM classifier to predict unseen test examples. Each training dataset contain 390 sequences (195 positive and 195 negative examples). The sequences were obtained from Fischer *et al. *and are available in Additional File 1.

#### 2. Test datasets

Test datasets were used for evaluating the performance of the SVM method. Each test dataset contains 48 sequences (24 positive and 24 negative examples). The sequences were obtained from recently discovered substrates extracted from literature search which were not reported in Fischer *et al*. Sequences are available in Additional File 2.

Datasets containing sequences identical to the P_4_P_1_, P_4_P_2_' and P_14_P_10_' datasets but without the P_1 _residue were also constructed (designated as P_4_P_1_(-D), P_4_P_2_'(-D) and P_14_P_10_'(-D) respectively). These datasets were divided into training and test datasets as mentioned earlier.

### Vector encoding schemes

To encapsulate the sequence information into a format suitable for SVM training and testing, the sequences were transformed into *n-*dimensional vectors using an orthonormal encoding scheme. Each amino acid is represented by a 20-dimensional vector, composed of either zero or one as elements. For example, alanine was represented as [0,0,0,0,0,0,0,0,0,0,0,0,0,0,0,0,0,0,0,1] and cysteine as [0,0,0,0,0,0,0,0,0,0,0,0,0,0,0,0,0,0,1,0]. Therefore, for the P_4_P_1 _dataset, each sequence was represented by an 80-dimensional vector. Sequences in the P_4_P_2_' and P_14_P_10_' datasets were represented by 120 and 480 dimensional vectors respectively.

### SVM implementation

For SVM implementation, we used the freely downloadable LIBSVM package by Chang and Lin [32]. Details of the SVM methodology can be obtained from the article by Burges [33]. Briefly, SVM is based on the structural risk minimization principle from statistical learning theory. A set of positively and negatively examples can be represented by the feature vectors *x*_*i *_(*i *= 1, 2,..., *N*) with corresponding labels *y*_*i *_∈ {+1,-1}. To classify the data, the SVM trains a classifier by mapping the input samples, using a kernel function in most cases, onto a high-dimensional space, and then seeking a separating hyperplane that differentiates the two classes with maximal margin and minimal error. The decision function for new predictions on unseen examples is given as:



where *K *(*x*_*i*_·*x*_*j *_) is the kernel function, and the parameters are determined by maximizing the following:



under the conditions,



The variable *C *serves as the regularization parameter that controls the trade-off between margin and classification error. As the efficacy of the SVM prediction system is dependent on the type of kernel used, we explored various kernels (linear, sigmoid, polynomial and the radial basis function) commonly implemented in biological problems on our datasets. We have chosen the widely used radial basis function (RBF) kernel as it was found to be most effective (data not shown):



Two parameters are required for optimizing the SVM classifier; *γ*, which determines the capacity of the RBF kernel and the regularization parameter *C*.

### SVM optimization

To optimize the SVM parameters *γ *and *C*, we applied 10-fold cross-validation on each of the training datasets using various combinations of *γ *and *C*. In 10-fold cross-validation, the training dataset was spilt into 10 subsets where one of the subsets was used as the test set while the other subsets were used for training the classifier. The trained classifier was tested using the test set. The process is repeated 10 times using a different subset for testing, hence ensuring that all subsets are used for both training and testing. SVM parameters *γ *and *C *were stepped through combinations of 0.01, 0.1, 1, 10, 100 for *γ*, and 1, 10, 100 and 1000 for *C *in a grid-based manner.

### SVM training and testing

The best combinations of *γ *and *C *obtained from the optimization process were used for training the SVM classifier using the entire training dataset. The SVM classifier was subsequently used to predict the test datasets. Various quantitative variables were obtained to measure the effectiveness of the SVM method:

(i) *TP*, true positives – the number of correctly classified cleavage sites.

(ii) *FP*, false positives – the number of incorrectly classified non-cleavage sites.

(iii) *TN*, true negatives – the number of correctly classified non-cleavage sites.

(iv) *FN*, false negatives – the number of incorrectly classified cleavage sites.

Using the variables above, a series of statistical metrics were computed to measure the effectiveness of the SVM method. *Sensitivity (SE) *and *Specificity (SP)*, which indicates the ability of the prediction system to correctly classify the cleavage and non-cleavage sites respectively, were calculated:



To provide an indication of the overall performance of the system, we computed *Accuracy (AC)*, for the percentage of correctly classified sites, and the *Matthews Correlation Coefficient (MCC)*.



### Prediction of caspase-mediated cleavage of Livin and mutants

The SVM trained using the P_14_P_10_' (-D) dataset (RBF kernel, *γ *= 0.1, *C *= 100) was used to predict the cleavage of Livin [Swiss-Prot:Q96CA5] and the various deletion mutants, based on the prediction of the caspase cleavage sites, as reported in Yan *et al*. [30]. 24 amino acids subsequence segments centred on the P_1 _residue of the reported Livin cleavage site (DHVD^52^) were extracted from both wild type and mutant Livin sequences. Mutants used in this study are: LE Δ52–61, Δ53–55, Δ55–57, Δ57–59, Δ60–62, Δ52–61, Δ53–61, Δ52 and Δ51–53. In mutants with Asp-52 deleted, the peptide windows were centred on the subsequent residue occupying position 52.

### Comparison with other available methods

As the CasPredictor method is unavailable from the published website, it was not tested. The performance of GrabCas was compared with the SVM method using the current datasets. As the GraBCas scoring matrices are specific for the tripeptide, P_4_-P_3_-P_2_, and assume that P_1 _is an Asp (D) residue, the GraBCas matrices were used to score only the positive sequences (cleavage sites) from the P_4_P_1 _training dataset. As GraBCas scores for different caspases were available, only the highest scores were recorded. The percentage of correctly predicted cleavage sites *(Sensitivity, SE) *were calculated as mentioned earlier. The P_4_P_1 _test dataset was tested in the similar manner and the *SE *score was obtained at a GraBCas cut-off of 0.1.

## Authors' contributions

LJKW conceived the application of SVM for prediction of caspase substrate cleavage sites. TWT contributed with ideas on the experimentation and SR finalized the manuscript. All authors read and approved the final manuscript.

## Supplementary Material

Additional File 1 Dataset of caspase substrate cleavage sites (for cross-validation and SVM training). List of caspase substrate cleavage sites used for cross-validation and training of the SVM. Click here for file

Additional File 2 Dataset of caspase substrate cleavage sites (for independent out-of-sample testing). List of caspase substrate cleavage sites used for independent out-of-sample testing of the SVM method. Click here for file

## Figures and Tables

**Table 1:** Results of SVM prediction for various test datasets.

**Test datasets**	***γ***	***C***	**Performance Evaluation**			
			
			**AC (%)**	**SE (%)**	**SP (%)**	**MCC**
**P**_**4**_**P**_**1**_	0.01	100	95.83	95.83	95.83	0.92
**P**_**4**_**P**_**2**_'	0.1	100	97.92	95.83	100.00	0.96
**P**_**14**_**P**_**10**_'	0.1	100	97.92	95.83	100.00	0.96
**P**_**4**_**P**_**1**_**(-D)**	0.01	1	81.25	62.50	100.00	0.67
**P**_**4**_**P**_**2**_'**(-D)**	1	100	89.58	79.17	100.00	0.81
**P**_**14**_**P**_**10**_'**(-D)**	0.1	1	93.75	87.50	100.00	0.88

The SVM parameters (*γ *and *C*) were obtained from the cross-validation conducted on the training datasets.

**Table 2:** GraBCas prediction on the P_4_P_1 _training dataset (positive sequences only)

**GraBCas Cutoff**	**SE (%)**
**0.1**	87.43
**1.0**	69.46
**5.0**	40.72
**10.0**	28.14
**20.0**	19.76

**Table 3:** SVM prediction of caspase substrate cleavage sites in Livin and mutants.

**Substrate**^*a*^	**Experimental Results**^*b*^	**SVM Prediction**^*c*^
Wild type Livin	Cleaved	Cleaved
LE Δ52–61	Not cleaved	Cleaved
Δ53–55	Cleaved	Cleaved
Δ55–57	Cleaved	Cleaved
Δ57–59	Cleaved	Cleaved
Δ60–62	Cleaved	Cleaved
Δ52–61	Not cleaved	Not cleaved
Δ53–61	Not cleaved	Cleaved
Δ52	Not cleaved	Not cleaved
Δ51–53	Not cleaved	Cleaved

*a*. Wild type Livin and various deletion mutants as reported in Yan *et al. b *Experimentally verified cleavage (*cleaved*) or non-cleavage (*not cleaved*) of Livin and deletion mutants. *c*. SVM prediction of caspase cleavage sites on Livin and deletion mutants (*Cleaved *– presence of cleavage site; *Not cleaved *-absence of cleavage site).
