# Supplementary material for: SVM-based prediction of caspase substrate cleavage sites
Source: BMC Bioinformatics. 2006 Dec 18;7(Suppl 5):S14. doi: 10.1186/1471-2105-7-S5-S14 (PMC1764470; doi:10.1186/1471-2105-7-S5-S14)
Supplement: Additional File 1 — Dataset of caspase substrate cleavage sites (for cross-validation and SVM training). List of caspase substrate cleavage sites used for cross-validation and training of the SVM. [file 1471-2105-7-S5-S14-S1.doc]

Dataset of caspase substrate cleavage sites (for cross-validation and SVM training).

| **Caspase Substrate** | **Uniprot Accession ID** | **Cleavage Site^1^** | **P_1_ Position^2^** |
| --- | --- | --- | --- |
|  |  |  |  |
| Acinus | Q9UKV3 | DELD | 1093 |
| Akt | P31749 | TVAD | 108 |
|  |  | EEMD | 119 |
|  |  | ECVD | 462 |
| α-Adducin | P35611 | DDSD | 633 |
| α-II-Fodrin | Q13813 | DETD | 1185 |
| Androgen Receptor | P10275 | DEDD | 155 |
| AP-2 α | P05549 | DRHD | 19 |
| Apaf-1 | O14727 | SVTD | 271 |
| APC | P25054 | DNID | 777 |
| Ataxin-3 | P54252 | LISD | 145 |
|  |  | LDED | 225 |
| ATM | Q13315 | DYPD | 863 |
| Bad | Q92934 | EQED | 14 |
| Bax | Q07812 | FIQD | 33 |
| Bcl-2 | P10415 | DAGD | 34 |
| Bcl-xL | Q07817 | HLAD | 61 |
|  |  | SSLD | 76 |
| β-Actin | P60709 | ELPD | 244 |
| β-Catenin | P35222 | TQFD | 115 |
|  |  | ADID | 83 |
|  |  | SYLD | 32 |
|  |  | YPVD | 751 |
|  |  | DLMD | 764 |
| β-II Spectrin | Q01082 | ETVD | 2146 |
|  |  | DEVD | 1457 |
| Bid | P55957 | LQTD | 60 |
| BLM | P54132 | TEVD | 415 |
| BRCA-1 | P38398 | DLLD | 1155 |
| BTF3 | P20290 | QSVD | 175 |
| c-Abl | P00519 | DTTD | 546 |
|  |  | DTAD | 655 |
| Calcineurin | P48452 | DGFD | 385 |
| Calsenilin | Q9Y2W7 | DSSD | 64 |
| Cas | Q63767 | DSPD | 748 |
|  |  | DVPD | 416 |
| CCT-α | P49585 | TEED | 28 |
| Cdc42 | P60953 | DLRD | 121 |
| Cdc6 | Q99741 | LVFD | 99 |
| CD-IC | O14576 | DSGD | 99 |
| c-FLIP | O15519 | LEVD | 376 |
| c-IAP1 | Q13490 | ENAD | 372 |
| Connexin 45.6 | P36383 | DEVE | 367 |
| CREB | P16220 | ILND | 140 |
|  |  | LSSD | 144 |
| CrmA | P07385 | LVAD | 303 |
| Cyclin A2 | P18606 | DEPD | 90 |
| Cytokeratin 18 | P05783 | DALD | 396 |
|  |  | VEVD | 237 |
| DCC | P43146 | LSVD | 1290 |
| Desmoglein-3 | P32926 | DYAD | 781 |
| E-cadherin | P12830 | DTRD | 750 |
| EGF-R | P00533 | DMDD | 1009 |
|  |  | DEED | 1006 |
| eIF2α | P05198 | DGDD | 303 |
| **Caspase Substrate** | **Uniprot Accession ID** | **Cleavage Site^1^** | **P_1_ Position^2^** |
| eIF3 | O75822 | DLAD | 242 |
| eIF4E-BP1 | Q13541 | VLGD | 24 |
| eIF4GI | Q04637 | DRLD | 1176 |
| Erb-2 | P04626 | SETD | 1125 |
| ETK/BMX | P51813 | DFPD | 242 |
| FAK | Q05397 | DQTD | 772 |
| FEM-1 | P17221 | ELLD | 320 |
| Ftase | P49354 | VSLD | 59 |
| γHSV68 Bcl-2 homolog | P89884 | DCVD | 31 |
| Gas2 | O43903 | SRVD | 278 |
| GATA-1 | P15976 | LSPD | 144 |
|  |  | EDLD | 125 |
|  |  | EGLD | 42 |
| GCL | P48506 | AVVD | 498 |
| Golgin 160 | Q08378 | ESPD | 59 |
|  |  | SEVD | 311 |
|  |  | CSTD | 139 |
| GRASP65 | Q91X51 | TLPD | 392 |
|  |  | SFPD | 374 |
| GrpL/Gads | O75791 | DIND | 241 |
| HEF1 | Q14511 | DLVD | 363 |
|  |  | DDYD | 630 |
| Helicad | Q8R5F7 | DNTD | 208 |
|  |  | SCTD | 251 |
| HIP-55 | Q6IAI8 | EHID | 361 |
| hnRNP A2/B1 | P22626 | VMRD | 55 |
|  |  | AEVD | 76 |
|  |  | KLTD | 49 |
| hnRNP C1/C2 | P07910 | EGED | 295 |
| hnRNP I | P26599 | LKTD | 139 |
| hnRNP R | O43390 | DYHD | 481 |
|  |  | KESD | 87 |
|  |  | DYYD | 472 |
|  |  | RAID | 66 |
| HPK-1 | Q92918 | DDVD | 385 |
| Huntingtin | P42858 | IVLD | 586 |
| ICAD | O00273 | DAVD | 224 |
| iPLA2 | O60733 | DVTD | 183 |
| KHSRP | Q92945 | QLED | 114 |
|  |  | EDGD | 116 |
|  |  | IGGD | 91 |
|  |  | AFAD | 76 |
|  |  | SQGD | 128 |
|  |  | IRKD | 72 |
|  |  | STPD | 102 |
| Lamin A | P02545 | VEID | 230 |
| LAP2-α | P42166 | SQHD | 482 |
|  |  | EERD | 440 |
|  |  | KRID | 412 |
| LEDGF | O75475 | DAQD | 486 |
|  |  | WEID | 85 |
|  |  | EVPD | 30 |
| Lyn | P07948 | DGVD | 17 |
| Max | P61244 | SAFD | 135 |
| Mcl-1 | Q07820 | EELD | 127 |
|  |  | TSTD | 157 |
| MEF2A | Q02078 | SSYD | 466 |
| **Caspase Substrate** | **Uniprot Accession ID** | **Cleavage Site^1^** | **P_1_ Position^2^** |
| MEF2D | Q14814 | LTED | 288 |
| MEKK1 | P53349 | DTVD | 874 |
| Mst1 | Q13043 | DEMD | 326 |
| Mst3 | Q9Y6E0-2 | AETD | 313 |
| Nedd4 | P46935 | DQPD | 237 |
| NF-kappa-B p65 | Q04206 | VFTD | 465 |
| NONO/p54nrb | Q15233 | MMPD | 421 |
| NP | Q701N7 | METD | 16 |
| Nucleolin | P19338 | AMED | 628 |
|  |  | TEID | 454 |
|  |  | GEID | 632 |
| NuMA | Q14980 | DSLD | 1726 |
| Nup153 | P49790 | DITD | 349 |
| p21Waf | P38936 | DHVD | 112 |
| p27Kip1 | P46527 | DPSD | 139 |
|  |  | ESQD | 108 |
| p28BAP31 | P51572 | AAVD | 163 |
| PA28γ | P61289 | DGLD | 80 |
| PAK2 | Q13177 | SHVD | 212 |
| PARG | Q86W56 | MDVD | 307 |
|  |  | DEID | 256 |
| Parkin | O60260 | LHTD | 126 |
| PARP-2 | O88554 | LQMD | 187 |
| Paxillin | Q8VI37 | SELD | 146 |
|  |  | SQLD | 301 |
|  |  | SLLD | 222 |
|  |  | FPAD | 165 |
|  |  | NTQD | 102 |
| PDE10A2 | Q9QYJ6 | DLFD | 315 |
| PDE6 | P16499 | DFVD | 166 |
| PIP5K-1α | Q99756 | DIPD | 279 |
| PKCδ | Q05655 | DMQD | 329 |
| PKCε | Q02156 | SSPD | 383 |
| PKCμ | Q15139 | CQND | 378 |
| PKCζ | Q05513 | DGMD | 239 |
|  |  | EETD | 210 |
| PKR | P19525 | DLPD | 251 |
| PLCγ1 | P19174 | AEPD | 770 |
| Plectin | Q15149 | ILRD | 2395 |
| Presenilin-1 | P49768 | AQRD | 345 |
| Presenilin-2 | P49810 | DSYD | 329 |
| pro-EMAP-II | P31230 | ASTD | 144 |
| pro-IL-1β | P01584 | YVHD | 116 |
| pro-IL-16 | Q14005 | SSTD | 510 |
| pro-IL-18 | Q14116 | LESD | 36 |
| Rad51 | Q06609 | DVLD | 187 |
| Ras-GAP | P20936 | DEGD | 157 |
| Rb | P06400 | DEAD | 886 |
| RET | P07949 | VSVD | 707 |
|  |  | DYLD | 1017 |
| RHA | Q08211 | EEVD | 167 |
| SATB1 | Q01826 | VEMD | 254 |
| SFRS1 | Q07955 | VYRD | 154 |
|  |  | DLKD | 138 |
|  |  | RKLD | 175 |
|  |  | CYAD | 150 |
| SLK | O54988 | DTQD | 436 |
| **Caspase Substrate** | **Uniprot Accession ID** | **Cleavage Site^1^** | **P_1_ Position^2^** |
| Sp1 | P08047 | NSPD | 590 |
| SS-B/La autoantigen | P05455 | DEHD | 371 |
| STAT1 | P42224 | MELD | 694 |
| TCRζ | P20963 | YLLD | 36 |
|  |  | GLLD | 28 |
|  |  | DTYD | 154 |
| TNF-R1 | P19438 | GELE | 260 |
| Topo I | P11387 | PEDD | 123 |
|  |  | EEED | 170 |
|  |  | DDAD | 146 |
| Tpr | P12270 | DESD | 2285 |
|  |  | DSQD | 1892 |
|  |  | DDGD | 2250 |
|  |  | DDED | 2117 |
| TRAF3 | Q13114 | ESVD | 368 |
|  |  | EEAD | 348 |
| U1-70-kDa snRNP | P08621 | DGPD | 341 |
| UFD2 | O95155 | VDVD | 123 |
|  |  | MDID | 109 |
| Vav-1 | P15498 | DQID | 150 |
|  |  | DLYD | 161 |
| Vimentin | P08670 | TNLD | 428 |
|  |  | IDVD | 258 |
|  |  | DSVD | 84 |
| vMLC | P08590 | DFVE | 134 |
| XIAP | P98170 | SESD | 242 |
|  |  |  |  |

**^1^** Cleavage sites are reported as tetrapeptides in the order: P4-P3-P2-P1. Except for DEVE (from connexin 45.6), GELE (fromTNF-R1) and DFVE (from vMLC), all cleavage sites have an Asp (D) in the P_1_ position.

**^2^** Indicate the position of the P_1_ amino acid in the protein sequence as reported in Uniprot.
