# Supplementary material for: SVM-based prediction of caspase substrate cleavage sites
Source: BMC Bioinformatics. 2006 Dec 18;7(Suppl 5):S14. doi: 10.1186/1471-2105-7-S5-S14 (PMC1764470; doi:10.1186/1471-2105-7-S5-S14)
Supplement: Additional File 2 — Dataset of caspase substrate cleavage sites (for independent out-of-sample testing). List of caspase substrate cleavage sites used for independent out-of-sample testing of the SVM method. [file 1471-2105-7-S5-S14-S2.doc]

Dataset of caspase substrate cleavage sites (for independent out-of-sample testing).

| **Caspase Substrate** | **Uniprot Accession ID** | **Cleavage Site^1^** | **P_1_ Position^2^** |
| --- | --- | --- | --- |
|  |  |  |  |
| 14-3-3 | P62258 | MQGD | 238 |
| AP-1 complex (γ-adaptin) | P22892 | DMTD | 746 |
| BAT3 | P46379 | DEQD | 1001 |
| CEACAM1-L | P31809 | DQRD | 460 |
| Claspin | Q9HAW4 | DEYD | 1072 |
| CTEN | Q8IZW8 | DSTD | 570 |
| DIAP1 | Q24306 | DQVD | 20 |
|  |  | VQPE | 205 |
| ERK2/MAPK | P63086 | ELDD | 334 |
| Her-2 | P04626 | DVFD | 1087 |
| JNK 1 β2 | P45983-4 | SDTD | 413 |
| MITF | O75030-9 | DLTD | 345 |
| NDUSF1 (p75 subunit of complex1) | P28331 | DVMD | 255 |
| Notch1 | P46531 | CLLD | 2193 |
|  |  | DCMD | 1874 |
|  |  | DHMD | 2095 |
| p23 co-chaperone | Q15185 | DGAD | 145 |
|  |  | PEVD | 142 |
| p65 | Q04206 | DCRD | 97 |
| PTEN | P60484 | DVSD | 371 |
|  |  | NEPD | 375 |
|  |  | QEID | 301 |
| Rad9 | Q6FI29 | DDID | 304 |
| SCL/Tal-1 | P17542 | EITD | 180 |
|  |  |  |  |

**^1^** Cleavage sites are reported as tetrapeptides in the order: P_4_-P_3_-P_2_-P_1_. Except for VQPE (from DIAP1), all cleavage sites have an Asp (D) in the P_1_ position.

**^2^** Indicates the position of the P_1_ amino acid in the protein sequence as reported in Uniprot.
